# Facile template-free synthesis of multifunctional 3D cellular carbon from edible rice paper†

**DOI:** 10.1039/d0ra01447h

**Published:** 2020-04-27

**Authors:** Monsur Islam, Peter G. Weidler, Stefan Heissler, Dario Mager, Jan G. Korvink

**Affiliations:** Institute for Microstructure Technology, Karlsruhe Institute of Technology Hermann-von-Helmholtz-Platz 1 76344 Eggenstein-Leopoldshafen Germany monsur.islam@kit.edu; Institute for Functional Interfaces, Karlsruhe Institute of Technology Hermann-von-Helmholtz-Platz 1 76344 Eggenstein-Leopoldshafen Germany

## Abstract

Edible rice paper wrapper is found to be an interesting precursor of a porous and light-weight carbon material. During pyrolysis, material samples show significant differences in length change, displaying typical 20–25% shrinking in the in-plane directions, and strongly expanding (up to 500%) across their out-of-plane direction. This results in a template-free synthesis of a 3D network of cellular carbon material. The out-of-plane expansion also allows for fabrication of 3D shapes of cellular carbon material from the 2D precursor. The rice paper derived carbon material features a hierarchical porosity, resulting in a specific surface area ranging from 6 m^2^ g^−1^ to 239 m^2^ g^−1^ depending on the synthesis temperature. The carbon material has a density of 0.02–0.03 g cm^−3^, and a higher modulus-density ratio than reported for other cellular carbon materials. It is mechanically stiff and exhibits excellent fire-resistant properties.

## Abstract 2

Edible rice paper wrapper is found to be an interesting precursor for template-free synthesis of lightweight, stiff, and fire-resistant 3D cellular carbon material.

## Graphical abstract

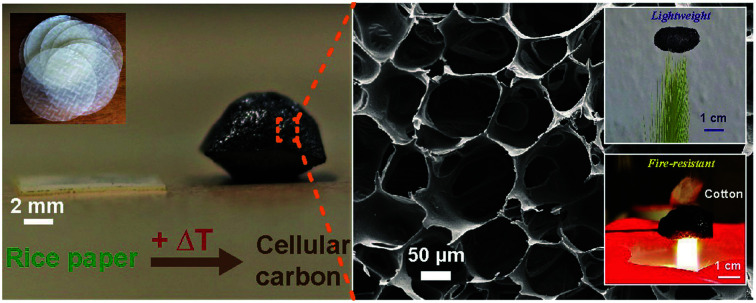

## 1 Introduction

Cellular carbon materials are interconnected three-dimensional networks of structural elements of carbon, which exhibit several attractive properties including low overall density, adjustable electrical and thermal conductivities, high specific mechanical properties, high chemical and thermal inertness, and high specific surface area.^1–3^ These properties have driven the use of cellular carbon materials, especially carbon foams, into a wide range of applications, such as electrodes for electrochemical cells,^4–7^ thermal insulators,^8,9^ catalyst support,^10,11^ and absorbents for oil and organic solvents.^12,13^ The precursors of the cellular carbons are abundant. Traditionally, cellular carbons are prepared by foaming of an oxygen-rich polymer (for example, polyurethane, furfural, resorcinol–formaldehyde, or phenol–formaldehyde) to form a cellular matrix of the precursor, followed by carbonization at a high temperature.^1,2^ A variety of biomasses have been also reported as precursors for cellular carbon materials, as these naturally feature a variety of cellular microstructures. Examples of such biomasses include wood, cotton, cellulose fibers, wheat flour, bread, and even fish skin.^14–19^

Even though the precursors are abundant, and wide range of cellular microstructures are available, the structuring of these materials into the required 3D shapes is still challenging. Traditional methods for 3D shaping of the cellular carbon mostly rely on molding of the cellular precursor. Fabrication of the mold can by itself be a challenge. Furthermore, clean demolding is an issue for several precursors.^20^ In recent years, only a few methods have been reported to fabricate 3D shapes of cellular carbon. In particular, additive manufacturing, including two-photon polymerization and stereolithography, has been reported to achieve 3D architectures featuring carbon micro-/nano-lattices.^21,22^ Although additive manufacturing approaches allow the fabrication of 3D complex shapes, also at the micro- and nano-scale, the process is still slow and relatively expensive. One of us recently reported on the fabrication of 3D shapes using origami folding, where we transformed a sheet of cellulose paper into a carbon 3D structure.^23^ Although this process allows for relatively fast and inexpensive fabrication of complex 3D designs, it still depends on the dexterity of the user. Consequently, the structural quality of the carbon origami depends on the user's skill as well. In all these methods, the precursor material act as a template for the cellular carbon, as the carbon inherits the cellular structure of the precursors during carbonization.

In this work, we present a facile and template-free method to synthesize cellular carbon using an edible rice paper wrapper feedstock as precursor. Rice paper, traditionally known as Bánh Tráng, is a popular ingredient in Vietnamese cuisine, and is popularly used as wrapping material in the preparation of finger foods and appetizers such as Vietnamese nem dishes. Traditionally, the paper is prepared by cooking of a thinly spread batter of rice flour mixed with a small quantity of tapioca starch, followed by cooling and sun drying.^24^ Commercially, it is available in the form of thin sheets. Since it is a consumer product, there are no detailed protocols about its production available, hence there might be differences in the result for rice papers from different vendors. Edible rice paper, to the best of our knowledge, has not been used before as a carbon precursor. Here, we present it as an interesting template-free precursor for achieving cellular carbon. We demonstrate the fabrication of 3D shapes of the cellular carbon material from in-plane shapes laser-cut from the rice paper. We characterize the material and microstructural properties of the resulting carbon material, and discuss the effect of these on the mechanical properties of the cellular carbon. Cellular carbon is mechanically stiff and exhibits excellent fire-resistant properties. We finish with a discussion on potential applications, with an emphasis on the material's multifunctionality.

## 2 Experimental

### 2.1 Materials

Commercially available rice paper wrappers (Brand: Thuong Hạng) was purchased from a local Asian grocery store. The rice paper was used as received for carbonization.

### 2.2 Shaping and carbonization of rice paper

Rice paper wrappers were cut into different 2D geometries using a CO_2_ laser cutting machine (ULS Versa Laser 3.50, wavelength: 10.6 μm). The patterned rice papers were placed in a tube furnace (Carbolite Gero FHA 13, Germany) for carbonization. The samples were carbonized using a heating protocol popularly used in the carbon microelectro-mechanical systems (C-MEMS) technology.^23,25,26^ Briefly, the heating protocol was: (i) heating from room temperature to a final temperature using a heating rate of 5 °C min^−1^; (ii) dwell at the elevated temperature for 1 hour; (iii) cooling down to room temperature by natural cooling, which was implemented by turning off the furnace. A constant nitrogen flow (0.8 L min^−1^) was maintained throughout the entire heating process. A final temperature ranging from 400 °C to 1200 °C was used for the carbonization process. The samples obtained after carbonization were named as RPC*X*, where *X* stands for the final temperature.

### 2.3 Material characterization

We characterized the microstructure of the carbonized samples using scanning electron microscopy (Carl Zeiss AG-SUPRA 60VP SEM). We measured the microstructural features such as width of large pores and strut diameter from the SEM images using ImageJ (https://imagej.net), a free image analysis software. To characterize the nature of the obtained carbon, Raman spectroscopy of the carbonized samples was performed on a Bruker Senterra set up (equipped with a confocal microscope) using a DPSS laser (*λ* = 532 nm) at 2 mW power with a penetration depth of <1 μm. The position, integrated area and full width at half maximum (FWHM) were calculated for each spectrum using the OriginPro software (https://originlab.com), using Lorentz profile to fit the first order regions. We performed X-ray diffraction (XRD) using a Bruker D8 Advance diffractometer using Cu-Kα_1,2_ radiation (*λ* = 1.5405 Å) to characterize the crystalline phases in our material. The porosity of the carbonized samples were determined by measuring the argon adsorption–desorption at 87 K using a Quantachrome Autosorb-1 MP gas sorption analyzer (Quantachrome Instruments, USA). The specific surface area of the carbonized samples were measured using Brunauer–Emmett–Teller (BET).^27^ Density function theory (DFT) model was used to determine the pore volume and pore size distribution.^28,29^

### 2.4 Mechanical properties

We calculated the structural density of the carbonized samples using the envelope method, *i.e.*, calculating the ratio between the mass and the volume of the carbonized samples.^30,31^ The mechanical properties of the carbonized samples were characterized by performing compression tests in an Instron Single Column Testing System (Model 4500). A load cell of 100 N and a compression rate of 1 mm min^−1^ were used for the compression tests.

### 2.5 Fire resistant properties

The RPC samples were soaked in IPA (VWR catalog number: 1.09634.1000) by putting the RPC samples in an isopropyl alcohol (IPA) bath. The weight of the IPA soaked RPC sample was measured using a weighing balance (Kern EG, KERN & Sohn Gmbh, Germany). The IPA soaked RPC samples were then set on fire using a candle flame. When the IPA was entirely burnt and no visible flame could be observed any longer, the weight was measured again. The mass fraction was calculated using eqn (1) and the absorption coefficient was calculated using eqn (2).1
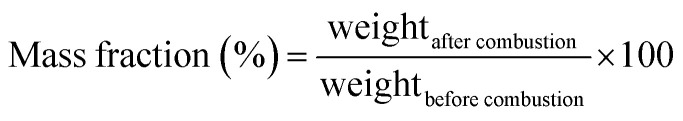
2
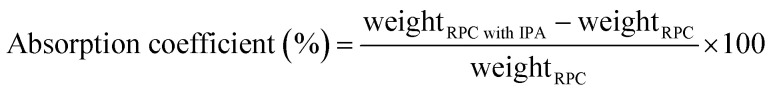


## 3 Results and discussion

### 3.1 Carbonization of rice paper

**Fig. 1:**
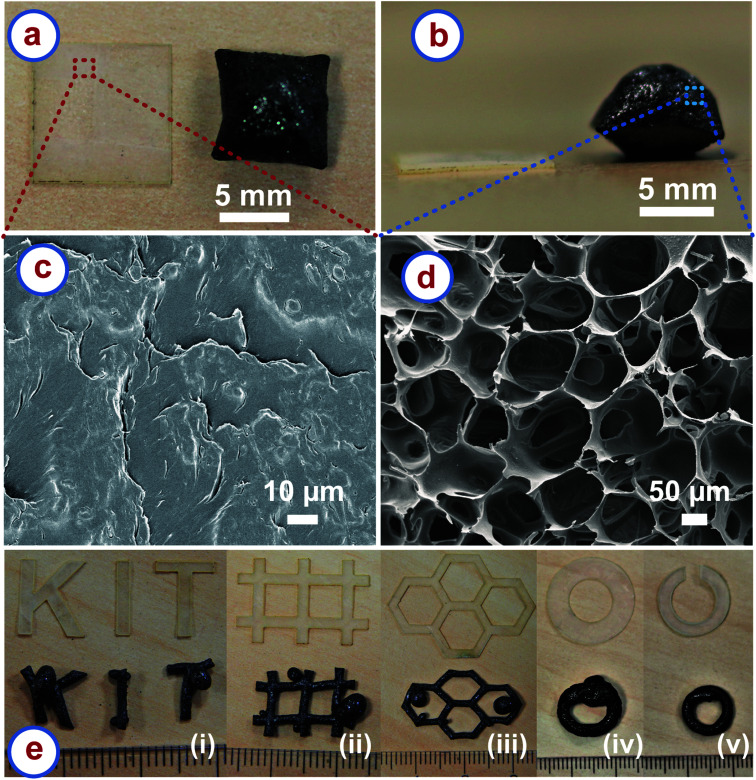
(a) Top view and (b) front view of a piece of rice paper, indicating the effect of carbonization. The top view shows the lateral shrinkage of the carbonized sample, whereas the front view shows that the carbonized sample features a significant increase in thickness. (c) SEM image of the rice paper showing a paste-like morphology. (d) SEM image of the carbonized rice paper showing an open-cell cellular microstructure, with pores at the 100 μm length scale. (e) (i–v) Different in-plane shapes of rice paper and their resulting 3D shapes obtained after carbonization. The rulers in the images indicate millimeters. The carbonization was carried out in a nitrogen environment at a temperature > 300 °C, with a heating rate of 5 °C min^−1^.

We pyrolyzed rice paper samples in a nitrogen environment at temperatures higher than 300 °C. Fig. 1a and b show the top view and the front view of a square piece of the rice paper before and after the carbonization, respectively. 20–25% shrinkage occurred in the lateral directions, which was expected as a characteristic of the carbonization process.^32^ However, to our surprise, a significant rise in the thickness of the carbonized sample was observed, which is uncommon during the carbonization process. The rise was highest at the middle of the carbonized samples, whereas it decreased towards the edges. The maximum rise at the middle ranged from 10 to 14 times of the thickness of the precursor rice paper leading to a volumetric expansion of around 500%. Such expansion behaviour was independent of the methods used for shaping the rice paper (*e.g.* laser cutting, scissors or tearing manually). The volumetric expansion of the rice paper during carbonization also resulted in a different microstructure than the precursor, when inspected using scanning electron microscopy (SEM). The precursor rice paper featured a morphology similar to a dried paste as shown in Fig. 1c. This was expected as preparation of rice paper includes cooking by heating of a thin spread batter of rice flour mixed with tapioca starch, followed by cooling and drying.^24^ In contrast, the carbonized rice paper exhibited a stochastic foam like microstructure featuring open cell structures, as presented in Fig. 1d. The distribution of the open cells was random, and the distant between the struts forming the open cells ranged from 12 μm to 250 μm as estimated from the SEM images.

We suspect the increase in the thickness and the evolution of a porous network might be result of a phenomenon similar to puffed rice preparation. During preparation of puffed rice, the rice is exposed to extreme heat, which causes rapid evaporation of water, and which builds up huge internal pressure in isolated pockets. This causes the rice expand to yield to a porous and puffy texture.^33,34^ Because rice paper is prepared from rice flour batter, the principal constituent material is also starch, known to be an excellent hydrophilic biopolymer.^35^ The hydrophilic nature of rice starch implies that the rice paper is expected to absorb a significant amount of moisture under normal ambient conditions. Similar to puffed rice, at the initial stage of heat treatment, the rice paper might have also experience rapid evaporation of the moisture, but without a free escape pathway, so that local steam-pockets act like a miniaturized pressure vessels. Furthermore, at high temperatures, the starch simultaneously softens.^33^ This might have led to puffing of the rice paper to release the built-up pressure to finally yield an enlarged, porous and dried precursor network. The carbonization process might have continued with such state of the precursor and finally resulted in the puffed, stochastic open-cell porous carbon material. However, this explanation is speculative. Further experiments are needed to validate this hypothesis.

One of the main advantages of the rice paper derived carbon (RPC) is that the puffing nature allows for conversion of a 2D shape of the precursor to a 3D shape of the cellular carbon. Fig. 1e shows different 2D shapes of the rice paper obtained using a laser cutting machine and their corresponding 3D shapes of the cellular carbon material obtained after carbonization. Apparently, bulged sections emerged at several places of the 3D shapes of the RPC samples, mainly at the corners. This might be to accommodate the extra volume produced during expansion of the material. We assumed that if some space could be freed up in the rice paper design, the expansion might be accommodated during carbonization. For example, Fig. 1e(iv) shows a disk of rice paper transformed into a 3D ring of carbon with a bulge. In the modified design, we cut a gap in the rice paper disk design to free up some space as shown in Fig. 1e(v). As anticipated, the split-ring disk resulted in a 3D carbon ring without any bulging segment after carbonization. We believe that the final shape can be controlled by anticipating the local degree of expansion, so that precise carbon shapes can be achieved. However, our method is only limited to shapes which are 3D projections of a 2D design, so-called Manhattan geometries. We cannot currently produce complex 3D structures as reported for additive manufacturing and origami techniques.^21–23,36^ Nevertheless, our method is a unique pathway for the fabrication of 3D shapes of cellular carbon material from 2D precursors. Unlike other methods, it does not require any 3D cellular precursor. Fabricating 3D cellular precursors can be cumbersome or challenging in many cases. For example, as mentioned earlier, carbon origami requires folding the precursor cellulose paper into 3D origami shapes, which depends on the dexterity of the user.^23^ Fabrication of carbon architectures using additive manufacturing requires fabrication of 3D architectures of the precursor polymer first, using a template method or direct writing method, which makes the whole process complicated.^21,22,36^ In comparison, here only planar shaping of the rice paper is required, which is possible by using inexpensive and fast processes such as laser cutting or cutting plotter machines.^37,38^ Hence, manufacturing is simple, fast, and inexpensive compared to the other processes. To the best of our knowledge, 2D shaping to achieve 3D topologies of cellular carbon has not been reported yet.

### 3.2 Material characterization of the rice paper derived carbon

**Fig. 2:**
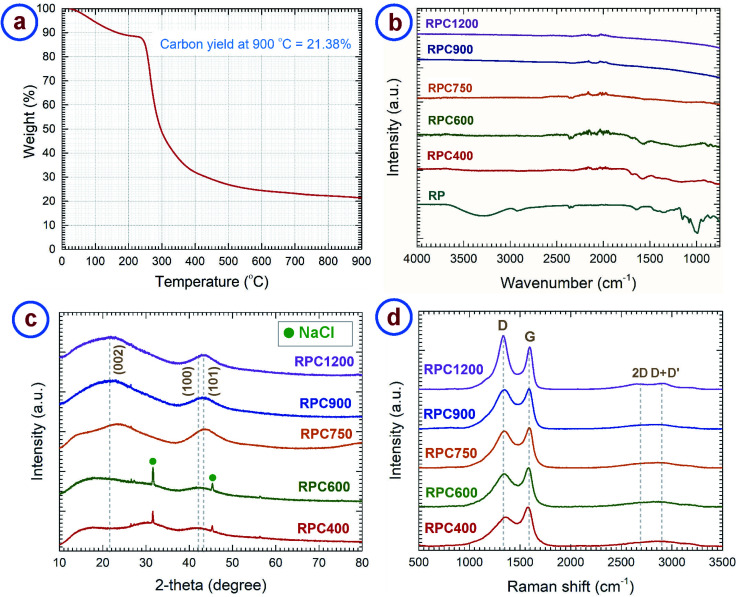
(a) Thermogravimetric analysis curve of rice paper performed under a nitrogen environment with a heating rate of 5 °C min^−1^. Spectral analysis: (b) FTIR, (c) XRD and (d) Raman spectra of rice paper derived carbon obtained at different temperatures. The carbonization process was carried out in a nitrogen environment with a heating rate of 5 °C min^−1^.

We investigated the material obtained through carbonization of the rice paper by different analytical methods. The thermogravimetric analysis (TGA) curve of the rice paper is presented in Fig. 2a, which shows the weight change of the rice paper during the carbonization process up to 900 °C. The TGA curve resembles the TGA curve of rice starch as reported in previous publications.^39,40^ The TGA curve exhibits three distinct regions. A weight loss of ∼13% was observed up to ∼240 °C, which can be attributed to the evaporation of the adsorbed and absorbed water from the rice paper. We assume the puffing of the rice paper to have occurred during this dehydration stage. A detailed later study of that phenomena might be worth to explore the possibility of fine tuning the degree of expansion. In a second stage, a rapid weight loss (∼54%) occurred in the range 240–380 °C. This was due to thermal decomposition of the constituent starch, and escape of the volatile byproducts.^41–43^ During the last stage, a gradual weight loss was observed for the temperature above 380 °C, which was due to the escape of heteroatoms such as hydrogen and oxygen. The carbon yield of the rice paper at 900 °C was 21.38%. This was slightly higher than the carbon yield of starch (15–17%) previously reported.^41^ We attribute this difference to the use of additives at unknown concentrations, including protein, oil, sugar and salt during the commercial production of rice paper.^24^

Fourier transform infrared (FTIR) spectroscopy of the rice paper and the carbon samples derived at different temperatures were performed to reveal the change in chemical structure of the carbon material with carbonization temperature. The results are shown in Fig. 2b. As expected, the rice paper exhibits FTIR spectra similar to starch.^44^ FTIR spectra of rice paper features major peaks for O–H vibrational stretching (3287.18 cm^−1^), C–H stretching (2925.99 cm^−1^), water adsorbed in the amorphous region of starch (1640.6 cm^−1^), –CH_2_OH vibrational stretching (1242.5.29 cm^−1^), and C–O–C ring vibrations (928.5 cm^−1^, 851.8 cm^−1^), which are the characteristic peaks for starch.^44–46^ These peaks disappeared in the FTIR spectra for the carbonized samples and peaks for aromatization of carbon were found instead. Carbonized samples RPC400, RPC600 and RPC750 °C showed characteristic peaks for aromatic C–H out-of-plane bending vibration (873.8 cm^−1^, 819 cm^−1^ and 749.2 cm^−1^), aromatic C=C and C–C vibrational stretching (1585.9 cm^−1^) and C=O vibrational stretching (1698.8 cm^−1^).^47–49^ However, intensities for all C–H bands decreased with an increase in temperature, especially the bands for RPC750 became extremely weak. This indicates the formation of graphene planes with increasing temperature. Upon further increase in temperature, the IR bands became nearly invisible, as seen in the FTIR spectra for RPC900 and RPC1200, although a faint aromatic C=C stretch band near 1585.9 cm^−1^ can be still observed in these spectra. This suggests the occurrence of complete decomposition of the side chains at a temperature in between 750 °C and 900 °C, and formation of turbostratic arrangements of the graphene sheets, which are inactive in the infrared.^50,51^

The results from FTIR were further supported by Raman spectra and X-ray diffraction (XRD) patterns. Fig. 2c shows the XRD patterns of the RPC samples. RPC750, RPC900 and RPC1200 show two broad maxima centering around 2*θ* = 22° and 2*θ* = 43°, which can be assigned to reflections of (002), and (100) and (101) planes, respectively, according to the International Centre for Diffraction Data (ICDD) PDF number 25-0284.^52–54^ Such XRD patterns confirm the turbostratic nature of the carbon derived from rice paper.^55–57^ However, the peak for the (002) plane was not evident in the XRD pattern of RPC400 and RPC600. The peak for the (100)/(101) plane was also considerably weak for these two samples. Furthermore, the integral width measured for the (100)/(101) peak decreased with the synthesis temperature as shown in Fig. S1a.† This supports the fact that, with increasing temperature, formation of graphitic planes of the carbon increases. In the diffractograms for RPC400 and RPC600, peaks for NaCl were also observed. These can be attributed to the salt added to the rice flour during preparation of the rice paper. We are not quite sure what might have happened to the NaCl during carbonization at temperatures between 600 °C and 750 °C, but no presence of NaCl was observed in the samples carbonized at 750 °C and higher.

The Raman spectra of the carbonized samples are presented in Fig. 2d. Two distinct characteristic peaks can be observed around 1350 cm^−1^ and 1585 cm^−1^. The peak around 1350 cm^−1^ is assigned to the D-band and corresponds to the A_1g_ breathing mode of the sp^2^ atoms in the ring at the *K*-point initiated from disorder.^58,59^ The peak around 1585 cm^−1^ is assigned to the G-band and corresponds to the zone center phonons of E_2g_ symmetry, responsible for the bond stretching of all the sp^2^ atoms in both rings and chains. Hence, the ratio between the intensities of the D-band and G-band (*i.e. I*_D_/*I*_G_) or the integrated areas of the D-band and G-band (*i.e. A*_D_/*A*_G_) represents the degree of graphitization.^58,60^ These ratios ideally decrease with graphitization and becomes zero for pure graphite. However, according to Ferrari and Robertson, this ratio increases with graphitization for amorphous and disordered carbon, as the peak for D-band arises from the aromatic rings.^58^ Such phenomena was observed in the case of the rice paper derived carbon samples. *I*_D_/*I*_G_ increases from 0.75 for RPC400 to 1.26 for RPC1200 (see Fig. S1b†). However, *A*_D_/*A*_G_ first increases from 2.42 for RPC600 to 2.85 for RPC900 and then decreases to 2.57 for RPC1200. The increase in *A*_D_/*A*_G_ till 900 °C can be attributed to the depolymerization of the rice paper, which results in more disordered carbon atoms in the matrix.^61–63^ As the depolymerization completes around 900 °C, a further increase in temperature results in the formation of ordered sp^2^ crystallites from the disordered carbon atoms, which results in a decrease in *A*_D_/*A*_G_ for higher temperature RPC samples. Increased ordering in the carbon structures can be also indicated by the decrease in the full width half maximum (FWHM) of the D-band with increased temperature as shown in Fig. S1c.†^63^

### 3.3 Microstructure and porosity of rice paper derived carbon

**Fig. 3:**
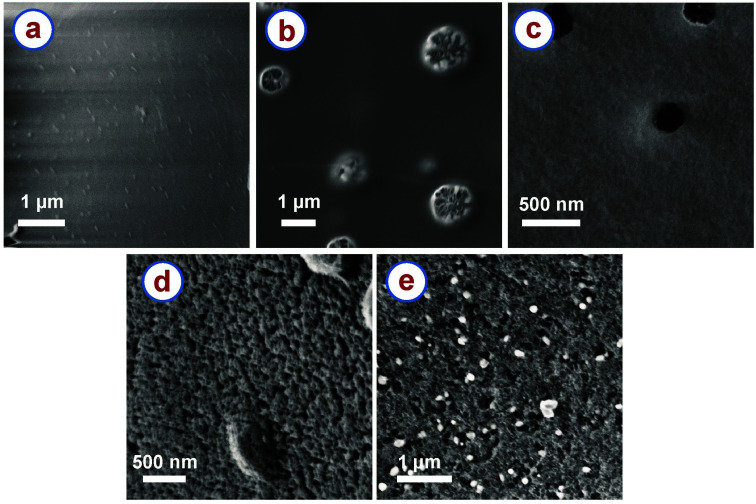
High magnification SEM images of rice paper derived carbon samples obtained at (a) 400 °C, (b) 600 °C, (c) 750 °C, (d) 900 °C and (e) 1200 °C.

The rice paper derived carbon samples featured an open cell cellular microstructure as shown in Fig. 1d. All the samples that were heat treated at a temperature higher than 300 °C featured such an open cell cellular microstructure. However, high magnification (>40k×) SEM images revealed that different surface textures were developed in the samples carbonized at different temperatures. Fig. 3 shows the surface textures of the RPC samples obtained after carbonization at different temperatures. These images show the evolution of internal pores and surface texture of the RPC samples with increasing temperature. RPC400 featured a smooth surface (Fig. 3a). RPC600 exhibited as well a smooth surface, although several pores started generating on the surface (Fig. 3b). Such pores were also seen in RPC750 as shown in Fig. 3c, however the surface looked relatively rough. As the carbonization temperature increased to 900 °C, a rougher surface was developed along with numerous tiny pores (Fig. 3d). Such evolution of pores in the temperature range 400 °C to 900 °C can be attributed to the escape of volatile carbonization byproducts from the material. In RPC1200, the surface morphology looked similar to RPC900. This was expected as no further generation of volatile byproducts is expected above 900 °C. However, the pores in RPC1200 seemed relatively larger when compared to RPC900 (Fig. 3e). We speculate that as the temperature increased above 900 °C, microstructural densification might have started due to rearrangement of the graphene planes, which might have caused collapsing of the tiny pores on each other forming relatively large pores.

**Fig. 4:**
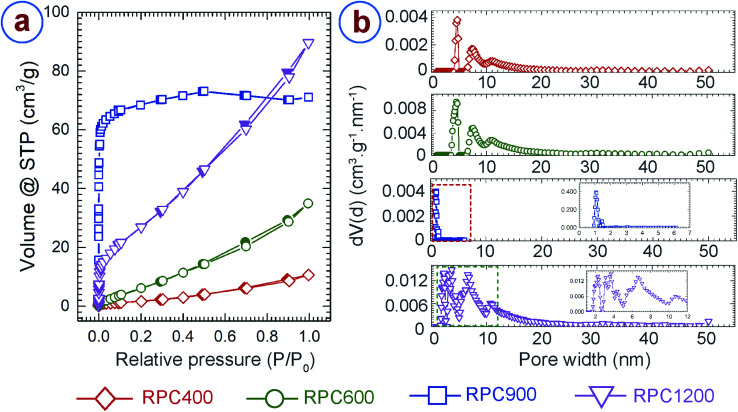
(a) Argon adsorption–desorption isotherm and (b) pore size distribution for the rice paper derived carbon samples. In the isotherms, the hollow symbols indicate adsorption and solid symbols represent desorption measurements. The pore size for RPC900 only ranged up to 7 nm, as indicated by the red squares in the pore size distribution plot for RPC900. The inset expands the pore size distribution of RPC900 in the marked region. The inset of the pore size distribution for RPC1200 expands the peaks in the region indicated by the dashed rectangle, for more clarity.

We further characterized the porosity and surface area of the RPC samples using argon adsorption–desorption,^28,64^ and the Brunauer–Emmett–Teller (BET)^27^ method, respectively. In addition, density functional theory (DFT) models were applied in order to determine porosity.^29^ Careful examination of the available DFT models revealed that the model using a mixture of cylindrical and spherical pore-shapes performed best. The adsorption–desorption isotherms and the pore size distributions for the RPC samples are presented in Fig. 4a and b, respectively. Both RPC400 and RPC600 exhibited an isotherm profile of type IV and hysteresis of H3 type, which suggests presence of mesopores (2 nm ≤ pore size ≤ 50 nm) in the sample.^28,64^ Both the samples exhibited an average pore diameter around 4.7 nm and 7.5 nm, as depicted from the respective pore size distribution plots. However, the gas adsorption was significantly low for both the samples, although the adsorption improved with synthesis temperature. This was expected as these two samples featured a very low number of internal pores as observed during morphological inspection of the samples using SEM. Due to lack of internal pores, RPC400 and RPC600 also resulted in low surface area, as shown in Table 1. The increase in the BET surface area and pore volume for RPC600 also confirmed the fact that more number of pores were evolved as the synthesis temperature increased. In contrast to RPC400 and RPC600, RPC900 and RPC1200 displayed much better adsorption and wider pore size distribution, which also yielded in significantly higher BET surface area and pore volume. RPC900 exhibited a type I isotherm profile, which is a characteristic for a microporous (pore size < 2 nm) material.^28,64^ This is further confirmed by the plot for pore size distribution showing dominance of pore sizes around 1.06 nm and 1.4 nm. Presence of mesopores was also indicated by the pore size distribution as the pore size in RPC900 ranges from 0.93 nm to 6.16 nm. Such a pore size distribution yielded to a total pore volume of 0.09 cm^3^ g^−1^ and a BET surface area of 239 m^2^ g^−1^. By comparison, RPC1200 exhibited an isotherm profile of type IV and hysteresis of H3 type, which suggests the formation of mesoporous structure.^28,64^ The plot for the pore size distribution for RPC1200 (inset of pore size distribution plot for RPC1200 in Fig. 4) confirms this resumé by displaying a dominance of pore sizes around 2.27 nm, 3.1 nm, 3.55 nm, 4.37 nm, 6.6 nm and 10.68 nm. The existence of few micropores and macropores (pore size > 50 nm) can also be surmized as the pore size ranges from 1.6 nm to 50.23 nm. The total pore volume of RPC1200 was 0.11 cm^3^ g^−1^. Such an increase in the pore volume for RPC1200, when compared to RPC900, can be attributed to the formation of a large number of mesopores. This also yielded to a BET surface area of 89 m^2^ g^−1^ for RPC1200, which is lower than that of RPC900. We speculated that, as the temperature increased above 900 °C, microstructural densification might have started due to rearrangement of the graphene planes, which might have caused merging of the tiny pores with each other, forming relatively large pores. Such phenomena resulted in lower BET surface area for RPC1200 than that of RPC900. Such decrease in BET surface area with increasing carbonization temperature was previously reported by other researchers when synthesizing activated carbon through pyrolysis of tomato waste and nutshells and herb residues.^65–67^ The BET surface area and pore volume obtained here are significantly lower than that of chemically activated carbon foams obtained from different other precursors.^68–71^ However, the BET surface area of our RPC samples are significantly higher than the commercially available cellular carbon materials, and comparable or even higher than the carbon foams obtained from the other precursors before activation.^68,70–72^ A much higher BET surface can be expected from the RPC samples, if activated using chemical activation methods.

**Table 1:** BET surface area and pore volume of rice paper derived carbon samples

Samples	BET surface area (m^2^ g^−1^)	Pore volume (cm^3^ g^−1^)
RPC400	6	0.01
RPC600	29	0.04
RPC900	239	0.09
RPC1200	89	0.11

### 3.4 Density and mechanical properties of rice paper derived carbon

**Fig. 5:**
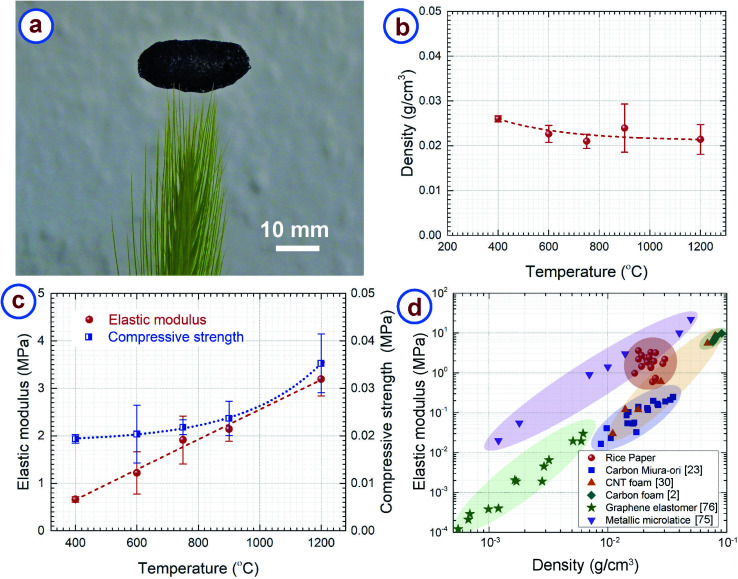
(a) The low density of rice paper derived carbon is demonstrated by balancing a sizeable sample on a grass stalk. (b) Effect of synthesis temperature on the structural density of the rice paper derived carbon. (c) Elastic modulus and compressive strength of rice paper derived carbon obtained at different temperatures. (d) Elastic modulus *vs.* density of rice paper derived carbon, compared with published data from other lightweight cellular materials.

The RPC samples featured very low density due to their hierarchically porous microstructure. Fig. 5a demonstrates the low density and resulting light weight of RPC samples. A macroscopically sized RPC sample was placed on the tip of a grass stalk without bending or buckling the strands of the grass. Fig. 5b presents the density of the RPC samples obtained at different temperatures, which are in the range 0.02–0.03 g cm^−3^, whereas the density of amorphous carbon is 2–2.3 g cm^−3^.^73,74^ Such low values can be attributed to the open cell porous structure of the RPC samples. The density does not change much with the synthesis temperature. As seen in the TGA of rice paper (Fig. 2a), around 10% of the weight loss occurred in the temperature range 400–1200 °C. The carbonization process simultaneously resulted in a volumetric expansion of ∼500%. We hypothesize that the high expansion overshadowed the weight loss of the material, which normally results in similar density in the temperature range used here. Nevertheless, a gradual decreasing trend can be seen for the density when plotting the best fit curve as shown in Fig. 5b.

The RPC samples exhibited a brittle-like nature during the compression tests. However, RPC400 and RPC600 seemed softer when compressing manually, whereas RPC1200 was the hardest of all the samples. Furthermore, during compression tests, at the point of failure, breakage of entire sample was observed for RPC400 and RPC600. In contrast, such a catastrophe was minimal in the other samples. For example, breakage was observed only at the elements of the cellular networks in contact with the load cell. The rest of the sample remained intact. This was reflected also in the values of compression strength and elastic modulus as shown in Fig. 5c. The compressive strength and elastic modulus increased with synthesis temperature. The compressive strength increased from 0.019 ± 0.001 MPa for RPC400 to 0.035 ± 0.006 MPa for RPC1200, and the elastic modulus increased from 0.662 ± 0.057 MPa for RPC400 to 3.194 ± 0.355 MPa for RPC1200. Our hypothesis is that RPC400 and RPC600 contained many different other materials (*e.g.* hydroxyl groups and carbonyl groups) other than pristine carbon as suggested by FTIR results, which might be responsible for the relatively soft nature of these samples. As the temperature increased, the other carbonaceous materials also transformed into pristine carbon, which means that the amount of pristine carbon increased with temperature, as did the sample homogeneity. This might be the reason the mechanical properties increased with temperature. It should be noted here that the standard deviation in the measurement of the mechanical properties was relatively large. This can be attributed to the random distribution of the pores and wide distribution of the open cell pore structures.

We plotted the elastic modulus of the RPC samples with other low density cellular materials for comparison in Fig. 5d. It can be seen that density of the RPC samples is comparable to metallic microlattices^75^ and carbon origami,^23^ and significantly lower than commercially available pitch derived carbon foams,^2^ although the density is still 2–3 times higher than that of CNT foams.^30^ The RPC samples exhibited elastic moduli higher than CNT foams and carbon origami, and comparable to metallic microlattices. The elastic modulus was also much higher than the projected elastic modulus of commercially available carbon foams^2^ and graphene elastomers^76^ at a similar density. Along with the structural properties, RPC samples are advantageous in terms of processing also, as synthesis of most of the cellular materials requires complex and expensive equipment. For example, processing of metallic microlattices involves photopolymerization of an epoxy polymer followed by electroless plating of metal on the epoxy structures.^75^ The epoxy was further chemically etched using a strong base solution. Furthermore, the synthesis of CNT foam relies on chemical vapor deposition growth of the CNTs,^30,77–79^ which is itself a complex and expensive process. Processing of carbon origami still requires dexterity of the user, hence the properties may vary depending on the individual. By comparison, synthesis of the RPC samples requires only one step pyrolysis and is completely independent of the user's skill. These make our process remarkably simple and inexpensive. It should be noted that the metallic microlattices and CNT foams exhibit a super-elastic characteristic by recovering the strain upon removal of compressive load, which is unlikely for our RPC samples due to its brittle nature.

### 3.5 Fire resistance of rice paper derived carbon

**Fig. 6:**
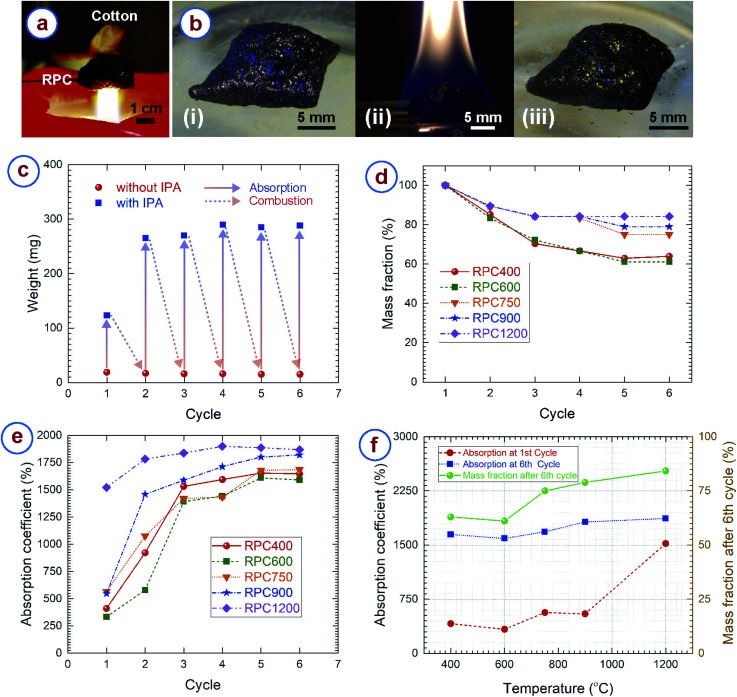
(a) Demonstration of the excellent fire resistant properties of the rice paper derived carbon sample showing that the cotton did not catch fire while residing on a rice paper derived carbon sample, when the other side of the carbon sample was in a direct contact with a candle flame. (b) Rice paper derived carbon sample (i) before soaking with IPA, (ii) during burning of the IPA, and (iii) after burning. The carbon sample remained unaltered during burning. (c) Example of the change of weight of a carbon sample after absorbing the IPA, and after combustion, for multiple cycles. This particular example is for a sample obtained at 900 °C. (d) Change of the mass and (e) absorption coefficient at different cycles for the carbon samples obtained at different temperatures. (f) Variation of the final mass, and initial and final absorption coefficient with carbonization temperature.

The cellular carbon samples obtained here showed excellent fire resistant properties. To demonstrate this, we held the RPC sample in a candle flame, and cotton fibers were attached to the opposite side of the RPC sample. The setup was maintained in the flame for at least 15 minutes. The temperature of candle flame can reach up to 1200 °C.^80,81^ However, the cotton (which spontaneously burns at around 300 °C (ref. 82)) never caught fire during the entire duration (Fig. 6a). Apart from confirming fire resistance, it also indicates the low thermal conductivity of the RPC samples, which can be attributed to the hierarchically porous microstructure of the RPC.^17^

For further characterization of the fire resistant properties, the RPC samples were soaked in isopropanol (IPA) and set on fire. As expected, the RPC samples remained unaltered during combustion and allowed to burn only the IPA (Fig. 6b). We repeated the experiments multiple times with the RPC samples obtained at different temperatures. The overall structure of the RPC samples remained unaltered after several cycles. Fig. 6c represents how mass of RPC samples changes after absorption and combustion of IPA at different cycles. It can be observed that after combustion the mass of the RPC samples remains almost unchanged. However, to our surprise, the absorbed mass increased significantly after the first cycle. To understand such behavior, we plotted the mass fractions and absorption coefficients at different cycles in Fig. 6d and e, respectively. We observed that the RPC samples experienced mass loss during the combustion, however the rate of mass loss was different for the RPC samples. The mass for RPC400 and RPC600 reduced to around 40% of its initial mass until the 5th cycle and remained unchanged after that. In comparison, 20% mass loss was observed for RPC750 and RPC900 till its 5th cycle, and afterwards no significant mass change was realized. For all these four samples, a considerable mass loss was realized until the 3rd cycle, and afterwards a gradual weight loss until the 5th cycle. In contrast, the mass loss for RPC1200 was only until the 3rd cycle, around 20% mass loss occurred during this period. Our hypothesis for the mass loss behaviour was that oxidation of the RPC samples might have occurred during combustion. However, we suspect that the oxidation might have happened due to presence of several functional groups in the carbon matrix. FTIR spectra of RPC samples showed that RPC400, RPC600 and RPC750 featured C–H and C=O groups (Fig. 2b), which decreased with carbonization temperature. Furthermore, we also speculate that localized temperature rise during combustion also might have occurred due to surface irregularities or localized material inhomogeneity. Such a localized temperature rise might have also caused localized oxidation of the RPC samples. SEM investigation of the RPC samples collected after combustion revealed that many new micro- and mesopores appeared in the material (Fig. S2c and d†). These new pores were identified by irregular shapes, and rough burnt edges, which were unlikely for the normal RPC samples. Furthermore, although the overall macroporous cellular microstructure was retained during combustion (Fig. S2a†), the edges of the struts appeared burnt and rough (Fig. S2b†). The localized oxidation might be the reason for the generation of the new pores and rough strut edges. However, these are speculations, further studies are needed for confirmation.

RPC400 and RPC600 exhibited an initial absorption coefficient around 300%, RPC750 and RPC900 exhibited an initial absorption coefficient around 500%, whereas RPC1200 exhibited an even higher absorption coefficient around 1500%. Fig. 6e shows that the absorption coefficient increases significantly after the first cycle for all the samples. The increase was stiff until the 3rd cycle for all the RPC samples except RPC1200. After the 3rd cycle, the increase was gradual and the absorption coefficient became consistent after the 5th cycle. In comparison, the increase in the absorption coefficient for RPC1200 was gradual and the absorption coefficient became almost constant after the 3rd cycle. The absorption coefficient increased from around 400% for the first cycle to around 1600% at the 6th cycle for RPC400 and RPC600; the increase for RPC750 and RPC900 was from ∼500% at the 1st cycle to ∼1700% and ∼1800%, respectively, at the 6th cycle (Fig. 6f). The absorption coefficient increased from ∼1500% at the 1st cycle to ∼1900% at the 6th cycle for RPC1200. Such a change in absorption coefficient directly correlates to the microstructure of the RPC samples collected after combustion after each cycle. As mentioned earlier, SEM images of the RPC samples collected after combustion showed the appearance of burnt and rough edges of the struts, and the generation of many new pores with burnt edges. Such rough edge struts and new pores are expected to increase the surface area of our samples significantly, which is advantageous for a variety of applications, including electrochemical energy devices and sensing materials. However, the generation of new pores might result in a decrease of the mechanical properties of the RPC samples, as the density is supposed to decrease due to the mass loss. Nevertheless, it should be mentioned here that the absorption the RPC samples exhibited was significantly higher than commercially available absorbent materials for organic solvents, and comparable to high absorption material reported earlier.^5,12,83–86^ The main disadvantage of our RPC samples are their brittle nature. This limits the recyclability of the absorbent RPC samples only through combustion, whereas other materials such as polymer foam or carbon aerogels also allow for recyclability through squeeze-recovery.^12,84^ However, it might be possible to recover the absorbed organic solvent by a distillation process as demonstrated by Li *et al.*^86^

## 4 Concluding remarks

In this work, we presented a facile and template-free strategy to fabricate lightweight, yet stiff and fire-resistant cellular carbon materials through the carbonization of sheets of edible rice paper. During carbonization, the rice paper went through a puffing process, which resulted in a volume expansion of ∼500%. This converted the 2D rice paper sheet to a 3D porous carbon material featuring open cell structures. The out-of-plane puffing nature of the rice paper allowed to fabricate 3D shapes of cellular carbon from corresponding 2D geometries of the precursor. This is extremely advantageous as, unlike for the traditional method, it does not require any cellular precursor template to fabricate the 3D shapes of the cellular carbon. The cellular carbon featured a hierarchically porous microstructure and a high BET surface area, which was significantly higher than commercially available carbon foams. The RPC samples featured a low density (∼1% of the density of amorphous carbon) and exhibited high mechanical stiffness at these low densities. Such mechanical stiffness was comparable or higher than that of other cellular materials such as metallic microlattices, carbon origami, CNT foams and carbon foams. The cellular carbon also exhibited excellent fire resistant properties and absorption of organic solvents.

We envision these carbon materials as multifunctional components in different applications. For example, integrating the mechanical stiffness and the fire-resistant properties, the RPC can be used as structural fire-protecting components for electronics. Furthermore, they can be promising for structural water filtering components from a water/organic solvent mixture. The multifunctionality can be expanded into different other areas as well. The use of cellular carbon materials for energy applications have been studied extensively by other researchers, due to their interesting electrochemical properties. In fact, porous carbon derived from starch, by itself or chemically activated, has been demonstrated for supercapacitors, photovoltaic deionization, and lithium–sulfur batteries.^87–89^ The electrochemical properties emanating from these studies combined with mechanical stiffness can lead to the fabrication of structural energy components, such as lightweight structural capacitors and batteries. The RPC can deliver the electrochemical energy, and simultaneously it will also be able to sustain a mechanical load in these applications. Furthermore, the ability to form 3D architectures from a 2D design of the precursor allows RPC for the fabrication of innovative and customizable 3D structures in these applications.

## Conflicts of interest

There are no conflicts to declare.

## Supplementary Material

RA-010-D0RA01447H-s001
