# Supplementary material for: Facile template-free synthesis of multifunctional 3D cellular carbon from edible rice paper
Source: RSC Adv. 2020 Apr 27;10(28):16616–28. doi: 10.1039/d0ra01447h (PMC9053079; doi:10.1039/d0ra01447h)
Supplement: RA-010-D0RA01447H-s001 [file RA-010-D0RA01447H-s001.pdf]

Electronic Supplementary Material (ESI) for RSC Advances.
This journal is © The Royal Society of Chemistry 2020

# Facile template-free synthesis of multifunctional 3D cellular carbon from edible rice paper

*Monsur Islam[a,]*, Peter G. Weidler[b], Stefan Heissler[b], Dario Mager[a] and Jan G. Korvink[a]*

[a]Institute for Microstructure Technology, Karlsruhe Institute of Technology, Hermann-von-Helmholtz-Platz 1, 76344 Eggenstein-Leopoldshafen, Germany

[b]Institute for Functional Interfaces, Karlsruhe Institute of Technology, Hermann-von-Helmholtz-Platz 1, 76344 Eggenstein-Leopoldshafen, Germany

*Corresponding author: monsur.islam@kit.edu

**Supporting Information:**

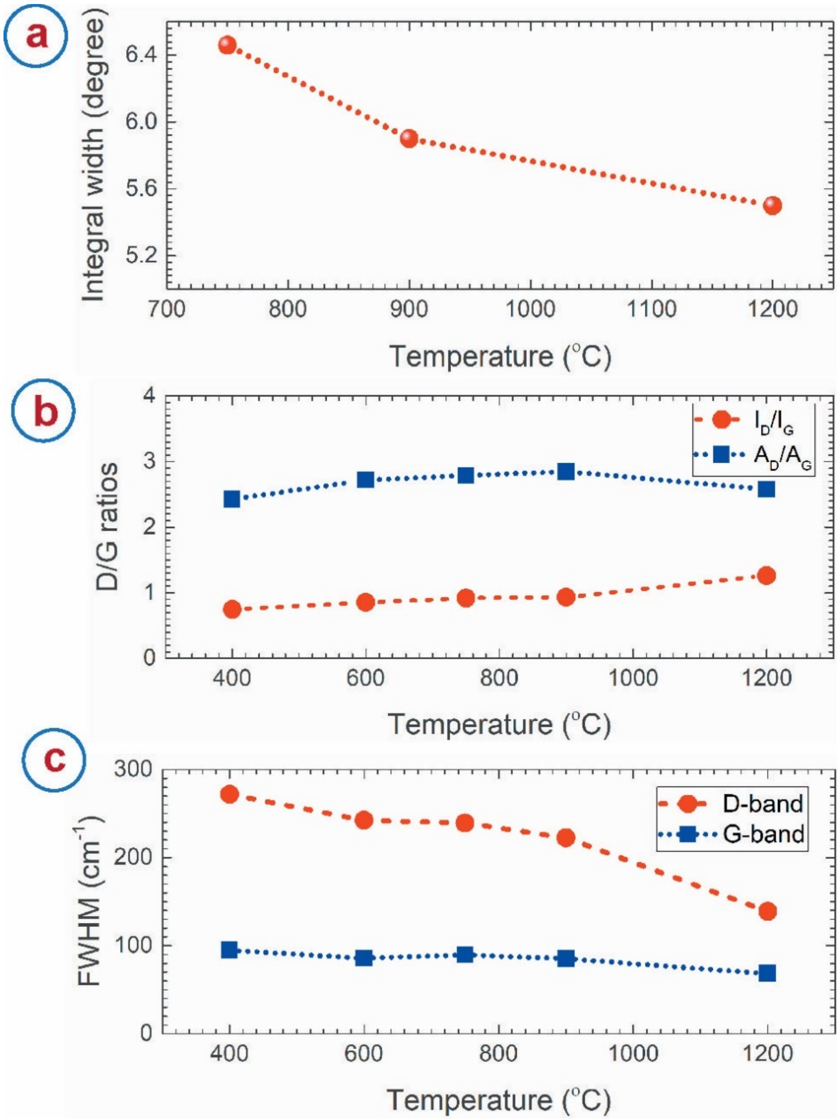


**Figure S1**: (a) Integral width measured for the peak at 2θ = 43° in the XRD patterns of the rice paper derived carbon samples at different temperature. (b) $I_D/I_G$ and $A_D/A_G$, and (c) full width half maximum (FWHM) of the D-band and G-band of the Rice Paper derived carbon (RPC) samples calculated using the Raman spectra.

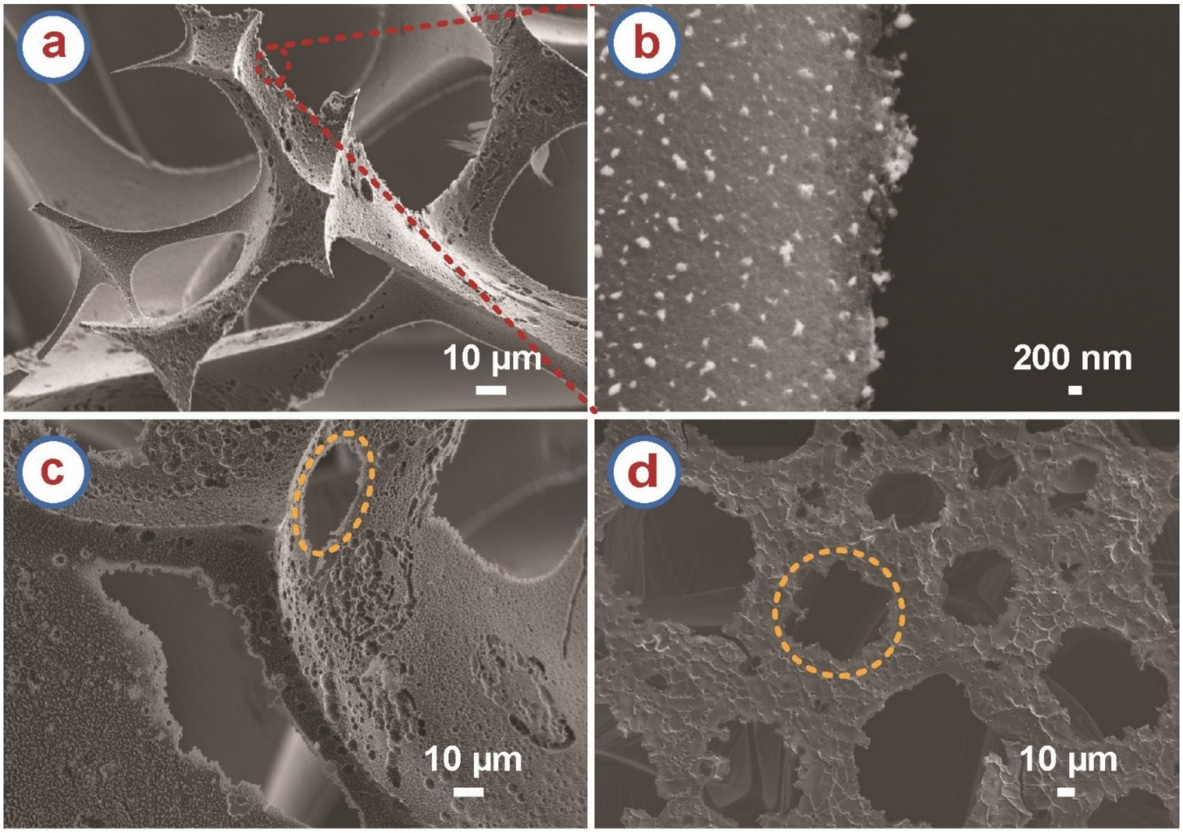


**Figure S2**: (a) Example of the microstructure of the RPC samples collected after the combustion test showing that the overall cellular macroporous microstructure was retained after the combustion test. (b) High magnification SEM image of the struts forming the cellular network, showing that rough edges were formed due to burning of the non-carbon compositions. (c) and (d) Examples of larger pores created due to burning.
